# Supplementary material for: Supraoptimal Cytokinin Content Inhibits Rice Seminal Root Growth by Reducing Root Meristem Size and Cell Length via Increased Ethylene Content
Source: Int J Mol Sci. 2018 Dec 14;19(12):4051. doi: 10.3390/ijms19124051 (PMC6321243; doi:10.3390/ijms19124051)
Supplement: Supplementary file 1 [file ijms-19-04051-s001.pdf]

Table S1. Primers used in the quantitative real-time PCR (qRT-PCR) analysis.

Gene name	Forward primer 5' → 3'	Reverse primer 5' → 3'
OsSAMS1	TTGACCGCAGTGGAGCATAC	ACGAATACGGACAGTGGCTC
OsSAMS2	CTTCGTCATCGGTGGACCTC	CTTGCAATGTAGGCTCCGCT
OsSAMS3	GGCCACATGTTCGGCTACG	GTGACCTGGGTCTTGCCGT
OsACS1	GCAGAGAGGGTACAAGGTGG	ACCCTGGGTAGTATGGGGTG
OsACS2	GAGGCATCCATTTGCACACC	TAAACTGGGCCATCGCCTTT
OsACS3	GAGGACCCCTATGACCCCAT	GGGTGCTCCCTCATGTATTCC
OsACS4	GATGTTGCGCTGGAGAGGAT	TGTTGCACATGCCCTTGGTA
OsACS5	GCTGGTTCAGGGTGTGCTTC	GCTTGTTGCTTTGTTCCATTCC
OsACO1	GGAGCAGCTGGATGATGCTT	CACACGCTTGTAGTGGTCCT
OsACO2	GCAGCATTGTCGTTCCCG	CGAGATGCCGTGGTTCAGG
OsACO3	AGAACTGGGGCTTCTTCGAG	AACTTCTCCTCCCGGCAGTT
OsACO4	GGTTTGAGGAGTGGGGGTTC	ACTGCGGGGTTGGATTCTTT
OsACO5	CGACAATGGCTGCGAGGAGT	CAAGCACACCTTCTTCACCCG
OsACO7	CCTGTAAGGACTGGGGCTTC	TCCAGGTGCTCGTCGTAATG
OsIAA3	GCCATGTTCCTCTGCTTCTCC	CGCCGTCCTTGTCCTCGTAG
OsXTH1	ACCGCCTACTACATGTGCTC	ATGATGTAGGGCTCACCCGT
OsXTH2	TGATCGCGTTCTTCGTGGAC	TCCACAGGCTGGAGTAGAGC
OsEXP3	ACATCGCCGTCTATCAAGCC	GTAGTCGTGACCGCTGATCG
OsEXP13	ACCAAAGGGTTCCTTGCATGA	ACTTGGAGCCCTTCACGTCC
OsEXPB4	CAGATGCACCAAGGACCAGT	GAGGTCGAAGTGGAACGGAG
OsEXPB11	AGGGTGCCATGCAAGTACAG	AGCACCTCGAAGTAGAACGG
OsActin	CTGACGGAGCGTGGTTACTCAT	TCATAGTCCAGGGCGATGTAGG
